# Experimental Demonstration of a Hybrid-Quantum-Emitter Producing Individual Entangled Photon Pairs in the Telecom Band

**DOI:** 10.1038/srep26680

**Published:** 2016-05-26

**Authors:** Geng Chen, Yang Zou, Wen-Hao Zhang, Zi-Huai Zhang, Zong-Quan Zhou, De-Yong He, Jian-Shun Tang, Bi-Heng Liu, Ying Yu, Guo-Wei Zha, Hai-Qiao Ni, Zhi-Chuan Niu, Yong-Jian Han, Chuan-Feng Li, Guang-Can Guo

**Affiliations:** 1 Key Laboratory of Quantum Information, University of Science and Technology of China, CAS, Hefei, Anhui 230026, China; 2 Synergetic Innovation Center of Quantum Information & Quantum Physics, University of Science and Technology of China, Hefei, Anhui 230026, China; 3 The state key laboratory for superlattices and microstructures, Institute of semiconductors, CAS, PO Box 912, Beijing 100083, China

## Abstract

Quantum emitters generating individual entangled photon pairs (IEPP) have significant fundamental advantages over schemes that suffer from multiple photon emission, or schemes that require post-selection techniques or the use of photon-number discriminating detectors. Quantum dots embedded within nanowires (QD-NWs) represent one of the most promising candidate for quantum emitters that provide a high collection efficiency of photons. However, a quantum emitter that generates IEPP in the telecom band is still an issue demanding a prompt solution. Here, we demonstrate in principle that IEPPs in the telecom band can be created by combining a single QD-NW and a nonlinear crystal waveguide. The QD-NW system serves as the single photon source, and the emitted visible single photons are split into IEPPs at approximately 1.55* μ*m through the process of spontaneous parametric down conversion (SPDC) in a periodically poled lithium niobate (PPLN) waveguide. The compatibility of the QD-PPLN interface is the determinant factor in constructing this novel hybrid-quantum-emitter (HQE). Benefiting from the desirable optical properties of QD-NWs and the extremely high nonlinear conversion efficiency of PPLN waveguides, we successfully generate IEPPs in the telecom band with the polarization degree of freedom. The entanglement of the generated photon pairs is confirmed by the entanglement witness. Our experiment paves the way to producing HQEs inheriting the advantages of multiple systems.

As reliable quantum emitters, single semiconductor QDs have been shown to be excellent building blocks in the fields of quantum computation^1^, quantum cryptography^2^ and quantum optics^3^. Single QDs’ desirable optical properties substantially meet the requirements of a single-photon emitter, including high-fidelity anti-bunching, narrow emission lines and high brightness. Moreover, they can potentially be integrated into monolithic structures, such as optical microcavities^4^,^5^ and electrical charge-tuning devices^6^. A popular realization involves Stranski-Krastanow InGaAs QDs embedded in a three-dimensional matrix^7^. However, extracting photons from a bulk semiconductor is highly inefficient because of the large mismatch in the refractive indices of GaAs and vacuum. To address this issue, an attractive approach is to embed the quantum dots in a nanowire^8^,^9^,^10^, which serves as a photonic waveguide. High coupling of the quantum emitter into the fundamental waveguide mode compared with higher order modes (*β*-factor ~1), thereby enabling near-unity light-extraction efficiency^11^. The only limitation preventing this QD-NW structure from use in practical quantum communication is its emission in the red, which is not suitable for long-distance transmission in an optical fiber.

Compared to single photons, IEPPs are considered more advanced tools for use in quantum information. IEPPs can function as single photons in all of the above-mentioned fields, but they are irreplaceable in long-distance quantum key distribution^12^, quantum teleportation^13^ and distributed quantum computation^14^. However, developing a quantum emitter that generates IEPPs remains a technological challenge. Recently, both polarization^15^ and time-bin entangled photon pairs^16^ were realized in a quantum dot system through a cascade radiation process. IEPPs in the telecom O band (1260–1360 nm) were also demonstrated utilizing an additional InGaAs strain relaxation layer to increase the emission wavelength of the quantum dot^17^. However, increasing the wavelength also increases the fine structure splitting, which allows only classical correlations to be observed. Moreover, similar to the single photon emitters based on a single QD, the wavelength tunability of the emission is extremely limited. To the best of our knowledge, generating IEPPs in the most commonly used ~1550 nm telecom band has not previously been demonstrated.

In contrast to a single QD, the wavelength of the photon pairs from the SPDC process can be directly tuned by changing the temperature or cut angle of the nonlinear crystal^18^. The SPDC process was used to generate entangled photon pairs in the telecom band^19^. However, the occurrence of the SPDC process is random; furthermore, it always has a finite probability of generating some multiple entangled pairs. Such multiple pairs induce errors and have a large negative impact on the rate of successful generating of long-distance entangled pairs. Even a small probability of generating multiple pairs will significantly reduce the efficiency when single photon detectors are employed^20^.

The zero multiple photon probability of single QD and the wavelength tunability of SPDC are prerequisites for producing an ideal IEPP emitter. Currently, as far as we know, there is no system that unifies these two properties. Principally, a hybrid system, i.e., the nonlinear crystal pumped by deterministic single photons, can evolve into a IEPP emitter. In previous systems, the nonlinear crystals always had to be pumped by a high-power laser due to the low conversion efficiency of the SPDC process. In nonlinear optical materials, such as *β*-barium borate (BBO) crystal, the efficiency is approximately 10^−11^ per pump photon^21^. However, technical progress has shown that it is possible to pump the nonlinear crystals by using a single-photon-level beam^22^,^23^,^24^. Major advances in nonlinear optics, such as the quasi-phase-matching of optical materials, have recently enabled access to higher inherent nonlinearities, such as that of the PPLN crystal. The downconversion efficiencies demonstrated in these materials can reach 10^−9^ in bulk^18^. The introduction of optical waveguides etched in a PPLN crystal has further increased the conversion efficiencies to 10^−6^ 25, thus enabling single-photon-level SPDC. In principle, the conversion efficiency can be further improved by increasing the length of the waveguide of PPLN. There is no physical limit to prevent the conversion efficiency from approaching the value of 1.

The existing techniques are adequate to fabricate an efficient single QD-NW and PPLN waveguide; however, the compatibility of a single QD-QW and PPLN is the main obstacle to producing this novel HQE. Generally, these limitations include matched emission wavelength, high-fidelity anti-bunching, narrow emission lines and high brightness. One can directly design a PPLN waveguide for a stable CW laser; however, for a single QD-NW it is quite another matter due to the uncertainty of both the emission wavelength and the optical quality. In our scheme, to generate non-degenerate IEEPs in the telecom band, the QD-NW emission should be as close to 775 nm as possible, resulting in the grestest wavelength tunability.

Using the current growth technique, it is not easy to control the QD emission to a certain wavelength with an accuracy of several nanometers. One feasible approach is to restrain the emission within a range of tens of nanometers covering the required wavelength. Based on the study of massive QD-NWs, several candidate QD-NWs can be selected at approximately 775 nm. Fine matching of the QD-NW and the PPLN waveguide can be realized through either tuning of the QD-NW (e.g., lateral strain or vertical electric field)^26^,^27^,^28^,^29^, or the temperature of the PPLN waveguide. Considering it is difficult to tuning the QD-NW while maintaining high single photon collection efficiency, temperature tuning of PPLN waveguide is more adoptable for this scheme. By assigning the emission wavelength of the QD-NW, one can design the PPLN waveguide that is most compatible with it; the details of this process are discussed below. Furthermore, a tradeoff exists between the brightness and linewidth for quantum emitters, i.e., a narrow linewidth corresponds to a long lifetime, which yields low photon counting rate. Due to the high *β*-factor, the reported QD-NW can realize >1 MHz count rate using a standard detector^11^,^30^. Another expected effect of QD-NW is luminescence linewidth broadening due to surface states^31^. To achieve efficient SPDC process, the linewidth of the pumping photons should be narrower than the PPLN waveguide conversion bandwidth.

In this work, we experimentally demonstrate a HQE generating IEPPs in the telecom band with the polarization degree of freedom. We fundamentally achieve the compatibility of the QD-PPLN interface, and the quantum witness result indicates the entanglement characteristic of the generated photon pairs. Our results are the first step toward distributing entanglement over a long distance^32^,^33^,^34^,^35^.

## Results

**Figure 1:**
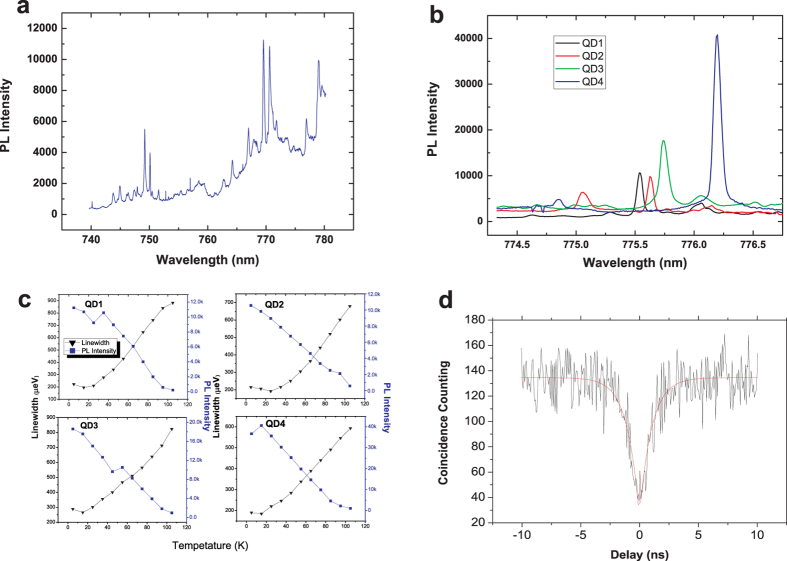
The optical characteristics of the QD-NW sample. (**a**) The PL spectrum of a QD assemble on one certain NW. (**b**) The PL spectrum of four candidate QD-NWs, the measuring temperature is 15 K. (**c**) The temperature dependence of the PL intensity and linewidth of four candidate QD-NWs. (**d**) HBT measurement result of QD4 emission using continuous wave above-band gap excitation. The error bars are smaller than the dimension of the markers and are not shown.

In this work, a newly designed QD-NW is used as the single photon emitter. The details of the growth methods and the micro-spectrum techniques are provided in the Methods section. The Photoluminescence (PL) study results show that the emission range of this QD-NW assemble is from 740 to 780 nm, which covers the target wavelength of 775 nm for our scheme, as shown in Fig. 1(a). To identify the most suitable QD-NW to achieve the highest compatibility of the PPLN waveguide, we narrow the search range within 10 nm around 775 nm. The PL of approximately one hundred possible candidate QD-NWs are investigated; four of them are shown in Fig. 1(b). For each of these four QDs, the temperature dependency of both emission intensity and linewidth are studied; the results are shown in Fig. 1(c). Tuning the cryostat temperature can achieve the largest *β*-factor, thus yielding a maximum counting of photons. The maximum counting points for these four QDs are 35 K, 95 K, 55 K and 15 K. The linewidth also substantially depend on the temperature and undergoes a narrowing trend, as previously reported^36^. It can be concluded that the narrowest points (15 K, 25 K, 15 K and 15 K) are generally not consistant with the maximum counting points. As a result, we have to determine the best balance temperature point that accounts for both the counting rate and the linewidth, instead of performing one-sided optimization. Comprehensively considering all of the desirable optical properties, QD4 emitting at 776.2 nm was selected as the single photon emitter to construct the HQE. The linewidth of this QD-NW is 185 *μ*eV at 15 K, which is significantly narrower than the conversion bandwidth of the PPLN waveguide (approximately 320 *μ*eV) applied in this experiment. The corresponding count rate is 3.3 MHz and the Hanbury Brown and Twiss (HBT) measurement shows a pronounced anti-bunching characteristic, with a multi-photon probability of *g*^2^(0) = 0.187 (Fig. 1d), which confirms that it is indeed a high purity single-photon source.

**Figure 2:**
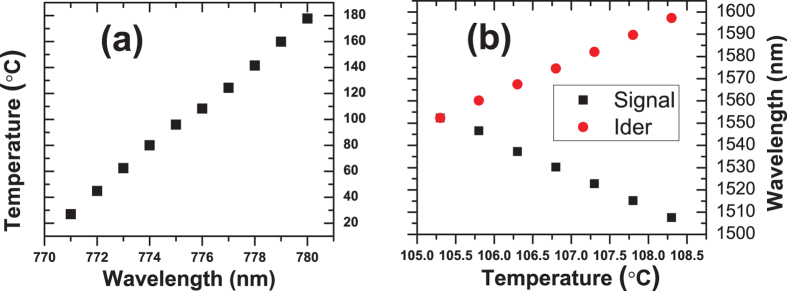
Experimental test results of the PPLN waveguide. (**a**) The degenerate down-conversion temperature points for varying pumping wavelength. (**b**) The wavelength-temperature dependence relationship of the telecom band photon pairs with 776.2 nm CW laser pumping. The error bars are smaller than the dimension of the markers and are not shown.

Using this QD-NW sample, the down-conversion characteristics of the PPLN waveguide are studied using theoretical calculation of two scenarios. First, the pump beam wavelength is changed and we calculate the temperature points to achieve degenerate down-conversion. Theoretical results show that, corresponding to 1 nm increasing on pumping beam, the temperature must be raised by 18.2 °C. The temperature controller can be tuned from room temperature to 200 °C, which restricts the feasible range to approximately 10 nanometers. In this scheme the PPLN feasible range should be from 770 to 780 nm, i.e., the occurrence of 775 nm down-conversion should be better at the centering of the tunable temperature range. Second, the pumping beam wavelength is fixed at 775 nm and we calculate the spectrum distribution of the IEPP. It can be concluded that the spectral separation increases with the temperature; the rate is measured as ~30 nm/°C. With these calculation results and considering that long crystals yielding more photon pairs and narrower bandwidths, we customized a 50-mm long flux-grown PPLN crystal with etched waveguides from HC Photonics with a grating period of 18.50 *μ*m for type-0 phase matching. The PPLN crystal is tested in the above two scenarios with a tunable cw laser resonant with the QD-NWs’ emission. As shown in Fig. 2, the measured results fundamentally fit with the theoretical calculations and the degenerate down-conversion point for 776.2 nm is 106.4 °C.

**Figure 3:**
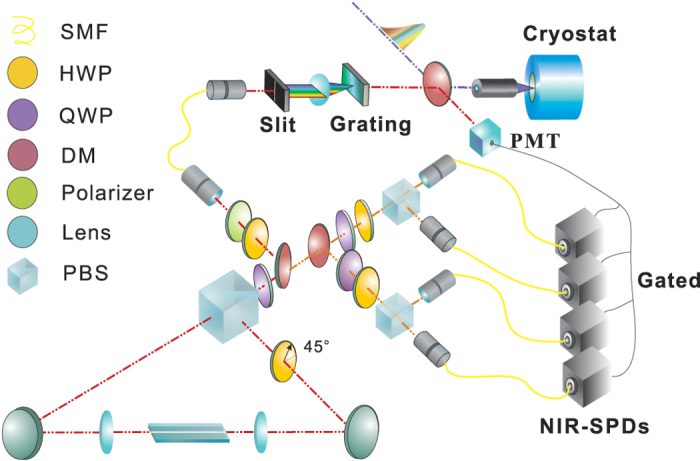
Experimental setup for the generation of IEPP of telecom band. The single QD-NW in the cryostat is excited by the pulsed ultraviolet (UV) beam and generates visible single photons with wavelengths of approximately 775 nm. The polarization Sagnac interferometer (PSI) apparatus with a PPLN waveguide at the center is used to generate polarization entanglement. The single photons coupled into the PPLN waveguide have a probability of splitting into IEPP, which are detected by near-infrared single-photon detectors (NIR-SPDs). The four NIR-SPDs are gated by the electrical pulses from the photo-multiple tube (PMT). SMF - single mode fiber, HWP - half wave plate, QWP - quarter wave plate, DM - dichroic mirror, PBS - polarized beam splitter.

As shown in Fig. 3, the experimental setup consists of two parts. The top-right portion is the single photon emitter, which uses a single QD-NW. The bottom-left portion is the entanglement generator based on the PPLN waveguide. After being filtered by the grating, the single photons are collected into a single mode fiber. The emitted single photons from the fiber are collimated by an achromatic lens. The polarization state is controlled by a combination of a polarizer and HWP. To generate the maximally entangled state, the photons’ polarization is set to the 45 degree diagonal state, written as 
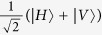
.

To separate the visible pump and telecom down-conversion photons, a dichroic mirror is applied to guide the visible pump photons into the entangled photon source setup, which is mainly a PSI. The bidirectional pumping of the crystal in counter-propagating directions creates a coherent superposition of the counter-propagating down-conversion outputs at the PBS, whose output photons are in polarization entangled state written as |*H*_*s*_*H*_*l*_〉 + *e*_*iϕ*_|*V*_*s*_*V*_*l*_〉. The interferometric combination of two outputs is typically sensitive to pathlength perturbations, however, the PSI configuration eliminates the need for path-length stabilization through its common path arrangement^37^,^38^. Moreover, the phase *ϕ* of the output state can be chosen by simply adjusting the relative phase between the horizontally and vertically polarized components of the pump. Details of the PSI and the entanglement generation process are provided in the Method section. The PPLN crystal was temperature stabilized at a phase-matching temperature of 107.3 °C above the degenerate point for 776.2 nm down-conversion, using a thermoelectric heater with a temperature stability better than 0.1 °C. The wavelengths of the signal and idler photons are centered at 1522.4 and 1583.7 nm, respectively. These telecom photons are split by a dichroic mirror. The two photons are measured by two standard polarization analyzers, either of which consists of a quarter wave plate (QWP), a half wave plate (HWP), a polarized beam splitter (PBS) and two near infrared single photon detectors (NIR-SPDs). The time stamps of all events were recorded when two photons were detected within 3 ns of each other by using a time-tagger with a resolution of 100 ps.

**Figure 4:**
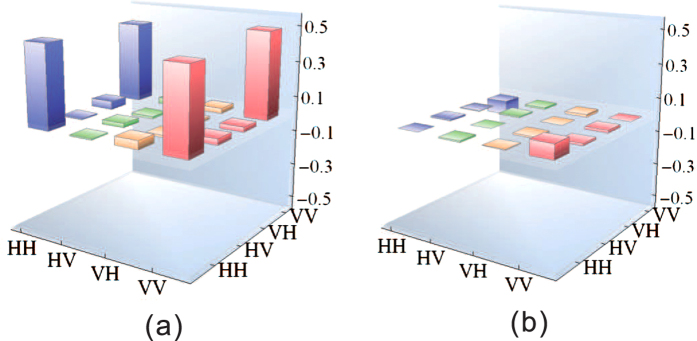
(**a**) Real and (**b**) Imaginary parts of the reconstructed density matrix of the PSI outcome state when the PPLN waveguide is pumped with a 776.2 nm continuous laser.

To verify that this setup is reliable for generating entanglement, first, a 776.2 nm continuous laser is adopted instead of the single photons to pump the PPLN waveguide. With a pumping power of 10 nW, we detect 3,000 pairs of telecom photons per second. The combined coupling and detection efficiency for the 1522.4 nm and 1583.7 nm photons is *η*1 = *η*2 = 0.08, as measured from the ratio of photon detections to coincident photon detections. The density matrix of the output state can be reconstructed by quantum state tomography. The coincidence counts recorded in 16 projective measurements can be used to obtain individual density matrix elements by using a maximum likelihood algorithm. The real and imaginary parts of the density matrix are plotted in Fig. 4. This density matrix estimation yields a level of fidelity of 0.936 ± 0.013 for the matching to a maximally entangled Bell state. The concurrence is measured to be 0.918 ± 0.011, which confirms that the PSI can generate high-quality entanglement.

For the single photon case with the QD-NW excited by the pulsed UV beam, approximately 0.6 M/s single photons are coupled into the PPLN waveguide for the SPDC process. The combined coupling and detection efficiency of the 1522.4 nm and 1583.7 nm photons is *η*1 = *η*2 = 0.036. The reduced efficiency likely results from the relatively long lifetime of the QD-NW (which is measured as 1.02 ns after fitting the HBT results), which leads to an exponential decay wave-package that cannot be completely covered by the 1-ns gate of the NIR-SPDs.

In contrast to quantum state tomography, the measurement of witness operators does not provide a complete reconstruction of the original quantum state, however, it does allow us to check the entanglement character of a quantum state by using a minimal number of local measurements for an^39^,^40^,^41^. The witness operator can be measured locally by select correlated measurement in the three complementary bases, which are linear(H/V), diagonal(D/J), and circular(R/L), with |*L*〉=
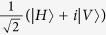
 and |*R*〉=
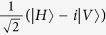
. To verify that the two photons are actually in an entangled state, the expectation value of the corresponding witness operator is expected to take a negative value. The entanglement witness is given by


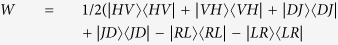


**Figure 5:**
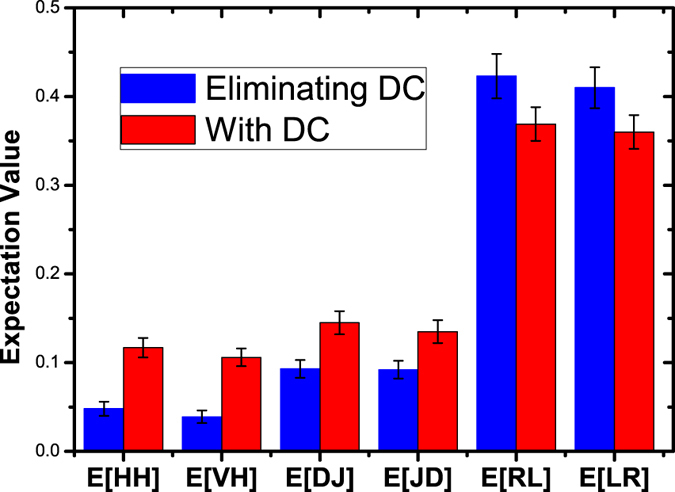
Experimental expectation values for every correlation function of the entanglement witness for the generated state. The results are derived by twofold coincidence measurements along three complementary common bases: linear(H/V), diagonal(+/−), and circular(R/L), conditioned by the pulsed signals from PMT. The blue bars are results after eliminating coincidence from the NIR-SPDs’ dark count, whereas the red bars include this noise.

There are four possible settings for each complementary base. A certain expectation value *E*[*ij*] (*i*, *j* = H, V, D, J, R, L) of a correlation function is obtained by making a von Neumann measurement along a specific basis and computing the probability over all the possible events. For example, for a HV correlation Tr(*ρ*HV), we perform measurements along the H/V basis. Next, its value E[HV] is given by the number of coincidence counts of HV over the sum of all coincidence counts of HH, HV, VH, and VV. Benefiting from the application of four NIR-SPDs, for each basis, the data for four settings can be recorded simultaneously. The experimental integration time for each basis is 35 h, and we record approximately 450 events of desired two-qubit coincidences for non-vanishing item, including the coincidence from the NIR-SPDs’ dark counts. To calculate W, six correlation functions are measured; their expectation values are shown in Fig. 5. The witness can then be directly evaluated as Tr(*ρ*W) = −0.11 ± 0.021. When the coincidence from the NIR-SPDs’ dark count is eliminated, the result is Tr(*ρ*W) = −0.28 ± 0.037. The error is obtained from Monte Carlo estimation. The negativity of the measured witness implies clearly that the telecom photon pairs are entangled. The imperfection of our data is due to the relatively low extinction ratio of the dual-wavelength devices and the PSI phase fluctuation during the long data recording time.

Our scheme provides a sub-Poissionian distribution of the IEPP in the telecom band that is inherited from the QD-NW emission. To further verify this result, the probability of multiple-photon-pairs is estimated by the autocorrelation *g*^2^(0) of either down-converted photon. The autocorrelation can be measured by analyzing the histogram of coincidence counts of the two NIR-SPDs in either polarization analyzer, and the generation of multiple-photon-pairs gives rise to photon coincidences at zero delay. The results for long and short wavelength photons are 0.176 and 0.182 respectively, when the coincidence from the NIR-SPDs dark count is eliminated. The fairly low values of *g*^2^(0) indicate sub-Poissonian distribution of the IEPP.

## Discussion

The main issues preventing our scheme from being applied in quantum communication are the dephasing of QD-NW and the low counting rate. On the one hand, the dephasing from QD can be dramatically suppressed by near-resonant excitation and coupling to a microcavity. Combining these two techniques single photons can be deterministically generated with extremely high single-photon purity and indistinguishability^42^. However, due to the difficulty in fabrication, the lack of microcavity is one main obstacle preventing QD-NW from high quality single photon emitter. Recently, ref. 11 integrated a gold mirror at the nanowire base to reflect downward-emitted photons and it is possible to form a microcavity with high-reflection coating fiber placed at the top the QD-NW^43^. On the other hand, there are several existing techniques to increase the IEPP count rate and each of the sections in our scheme can likely be improved. First, the coupling efficiency of the QD-NW emission can still be significantly increased by configuring the emission direction. Chu *et al*. designed an optical antenna to convert the dipolar radiation of an arbitrarily oriented quantum emitter to a directional beam with an efficiency more than 99%^44^. Second, in a periodically poled waveguide with high-reflectivity dielectric mirrors deposited on the waveguide end faces, the SPDC can be significantly enhanced at the resonances of the cavity^45^. Third, with the modern superconducting NIR-SPDs, the detection efficiency can be close to unity^46^. For any of these improvements, the IEPP count rate can be raised by at least one order of magnitude. Furthermore, the wavelength tunability of our scheme makes it more flexible for multi-user quantum communications. A programmable temperature controller for the PPLN waveguide can enable the compatibility with quantum wavelength-division multiplexing (WDM)^47^.

In summary, to the best of our knowledge, for the first time a HQE was experimentally demonstrated to generate IEPPs in the telecom band. The QD-NW employed in this experiment is a uniquely designed single-photon source and has a extremely high luminescence efficiency. The 50-mm-long PPLN waveguide used here possesses the highest conversion efficiency in SPDC process. A QD-NW meeting all of the desirable conditions for the QD-PPLN interface is found to construct this HQE. The quantum witness results show that the generated photon pairs are strongly entangled. Our scheme is the first step toward the fabrication of a practical IEPP source in the telecom band.

## Methods

### Single photon emitter

The formation of the QD-NW utilizes epitaxial growth, that is, “bottom-up” approaches, including the vapor-liquid-solid (VLS)^48^ and selective area epitaxy methods. This type of QD-NW system has also been proven to be a better gain medium than the homogeneous systems^49^,^50^. The QD-NW structure consists of a GaAs core, and a single separated GaAs QD can be achieved by using single *Al*_0.58_*Ga*_0.42_*As* quantum ring (QR) on the facet of a NW as the bottom surrounding barrier and an *Al*_0.7_*Ga*_0.3_*As* shell as the top barrier, which serves as single-photon emission source. This QD-NW sample is placed in a cryostat and cooled down with liquid Helium. In the PL study, the pump beam uses the 632.8 nm line of a cw He-Ne laser, which is focused on the sample through a confocal microscopy system. With one piece of a 1,200 groove/mm grating, followed by a lens and slit. Single emission lines from QD-NWs can be isolated to implement the HBT measurement.

For the generation of IEPPs in the telecom band, the QD-NW is excited with a pulsed uv pump centered at 388.1 nm. This UV beam was generated through the secondary harmonic generation (SHG) process in a BBO crystal pumped by femtosecond 776.2 nm pulsed laser, at a repetition rate of 76 MHz. After the SHG process, the light passes through a DM, that is highly transmissive at 776.2 nm and highly reflective at 388.1 nm, and the residual 776.2 nm light is transmitted to a photo-multiple tube (PMT). The generated electronic pulses from the PMT are used to trigger the four NIR-SPDs. The reflected UV pulses excite the QD-NW to generate single photons.

### PSI and entanglement generation

The PSI is composed of two silver mirrors and a PBS that serves as the input and output optical element. A PPLN waveguide for generating entangled photons by SPDC is located within the interferometer. The Sagnac configuration calls for an input-output PBS that operates at both the pump and down-conversion wavelengths. Instead, we used a commercially available broad band PBS cube. The PBS provides acceptable extinction ratio of (150:1) and (35:1) for the visible pump and telecom photon pair, respectively. The PBS serves to produce counter-propagating pumps for the bidirectional pumping of the down-conversion crystal. The 50-mm PPLN crystal is mounted in a crystal oven at the center of the PSI. For dual-wavelength capability, the PPLN crystal is uncovered without any tail optical fiber. The pumping single photons are coupled to the waveguide directly using a pair of aspheric lenses. These f = 11.17 mm coupling aspheric lens are customized with a dual-color AR coating. A dual-wavelength half-wave plate (DW-HWP) designed for both 776.2 nm and the telecom band rotates the V-polarized pump to the H polarization that is required (along the crystal’s y axis) for type-0 phase matching. It creates non-degenerate photon pairs with identical polarizations 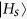
_↻_ and 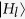
_↻_, that propagate in clockwise direction. The subscripts s and l denote the short and long wavelengths centered at 1522.4 and 1583.7 nm, respectively. The horizontal component of the beam is transmitted through the PBS and creates pairs 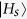
_↺_ and 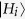
_↺_, propagating in the counterclockwise direction; these pairs are flipped to 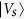
_↺_ and 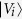
_↺_, respectively, by the DW-HWP. A small amount of both the 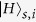 and 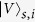 components leak from the idle exit port of the low extinction ratio PBS; however, the balance between these two components can be maintained by adjusting the pump polarization slightly. The counterpropagating pairs in the ↻ and ↺ modes are then combined at the PBS, where again the horizontal photons are transmitted and the vertical photons are reflected such that the outcome state is 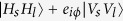.

## Additional Information

**How to cite this article**: Chen, G. *et al*. Experimental Demonstration of a Hybrid-Quantum-Emitter Producing Individual Entangled Photon Pairs in the Telecom Band. *Sci. Rep*. **6**, 26680; doi: 10.1038/srep26680 (2016).
